# C-reactive protein-triglyceride glucose index is a reliable biomarker for osteoarthritis: A cross-sectional study based on NHANES 1999–2018

**DOI:** 10.1097/MD.0000000000048314

**Published:** 2026-04-17

**Authors:** Rong Xie, Juncheng Long, Dongguang Liu

**Affiliations:** a Department of Orthopedics, The First School of Clinical Medicine, Guangdong Medical University, Zhanjiang, Guangdong, China; b Department of Orthopedics, The Affiliated Yangjiang Hospital, Guangdong Medical University (People’s Hospital of Yangjiang), Yangjiang, Guangdong, China.

**Keywords:** C-reactive protein-triglyceride glucose index, inflammation, insulin resistance, mortality, NHANES, osteoarthritis

## Abstract

Osteoarthritis (OA) is a prevalent degenerative joint disease with strong links to chronic inflammation and metabolic risk factors, yet comprehensive biomarkers for early risk stratification and prognosis are still lacking. The C-reactive protein-triglyceride-glucose index (CTI), as a novel composite marker reflecting both insulin resistance and systemic inflammation, may serve as a useful tool for OA risk assessment and management. We analyzed data from 10,372 participants of the National Health and Nutrition Examination Survey (NHANES) conducted between 1999 and 2018. The association between CTI and the prevalence of OA as well as all-cause mortality was assessed using multivariate logistic regression models, Cox regression models, restricted cubic spline functions, and receiver operating characteristic curves. CTI was significantly associated with OA prevalence (fully adjusted OR = 1.35, 95% CI: 1.21–1.50, *P* < .001) and showed better predictive performance than either C-reactive protein or triglyceride-glucose index alone. Among the 1064 OA patients, elevated CTI values were independently associated with increased all-cause mortality (HR = 1.24, 95% CI: 1.03–1.50, *P* = .02), demonstrating a clear dose-response relationship. In this cross-sectional study, CTI was strongly associated with the prevalence of OA and all-cause mortality in patients with OA. However, causality could not be inferred from these observational data.

## 1. Introduction

Osteoarthritis (OA) is a common yet complex degenerative joint disease that affects nearly every joint in the human body, with the knee and hip joints being the most commonly affected. It is the leading cause of disability worldwide.^[1]^ According to epidemiological studies, approximately 7% of the global population is affected by this disease, which not only significantly impairs the quality of life but also imposes a severe economic burden on both individuals and society.^[2]^ OA has long been considered to be a purely degenerative condition. However, although excessive joint loading is a major contributor to OA, it cannot account for the elevated risk of OA in non-weight-bearing joints such as the hands and wrists. This implies that the impact on OA risk is systemic rather than purely mechanical.^[3]^ Recent research findings have also demonstrated that the progression mechanism of OA is complex and involves multiple factors including mechanical, inflammatory, and metabolic processes.^[4]^ In healthy joints, chondrocytes maintain a stable metabolic equilibrium.^[5]^ In contrast, OA lesions induce a shift in chondrocyte metabolic status, transforming them from a resting state to a highly metabolically active state, with impaired metabolic flexibility.^[6,7]^ At the same time, chronic low-grade inflammation is also a key factor in the onset and progression of OA.^[8]^ Consequently, the “metabolic phenotype” of OA and the role of chronic inflammation are attracting increasing research attention.

C-reactive protein (CRP) serves as a commonly used systemic inflammatory marker, whereas the triglyceride-glucose (TyG) index acts as a surrogate marker for insulin resistance – a core driver of multiple metabolic disorders. Both have been demonstrated to be independently associated with an increased risk of OA.^[9-11]^ However, a single biomarker may not fully capture the complex interactions between the inflammatory process and metabolic dysfunction. The C-reactive protein-triglyceride glucose index (CTI) is a novel composite biomarker integrating CRP and the TyG index, first proposed in 2022.^[12]^ Due to its combination of inflammatory and metabolic components, it has demonstrated promising application value in the progression and outcomes of reproductive-related diseases, cardiovascular and cerebrovascular diseases, and cancers.^[13-17]^

Current treatment strategies for OA primarily focus on alleviating pain and performing total joint arthroplasty.^[18]^ Therefore, early identification and proactive interventions targeting modifiable risk indicators are crucial for prevention. At the same time, given closely associated with inflammation and insulin resistance, which are also major contributors to premature mortality.^[19,20]^ Therefore, identifying the comprehensive biomarker that reflects both inflammatory and insulin resistance states may be useful for stratifying outcomes in patients with OA. In summary, the primary objective of this study is to investigate the association between CTI and OA prevalence, and further evaluate its role in stratifying all-cause mortality risk among patients with OA.

## 2. Materials and methods

### 2.1. Study population

This study utilized data from ten consecutive 2-year cycles (1999–2000 to 2017–2018) of the National Health and Nutrition Examination Survey (NHANES) to conduct an in-depth analysis of the relationship between the CTI and OA. The NHANES is a nationally representative health survey conducted by the National Center for Health Statistics (NCHS). It employs a complex multistage stratified probability design to collect data from a representative sample of the US noninstitutionalized population. This survey combined interviews and physical examinations conducted by experienced medical personnel to collect data on sociodemographic characteristics, physical examination findings, nutritional status indicators, laboratory tests, and overall health status.^[21]^ Conducted every 2 years, it aims to assess the correlation between nutrition, disease prevention, and health promotion.^[22]^ All NHANES protocols were approved by the NCHS Institutional Review Board, and all participants voluntarily provided written informed consent. Further details regarding the NHANES database are available at https://www.cdc.gov/nchs/nhanes.

**Figure 1.**
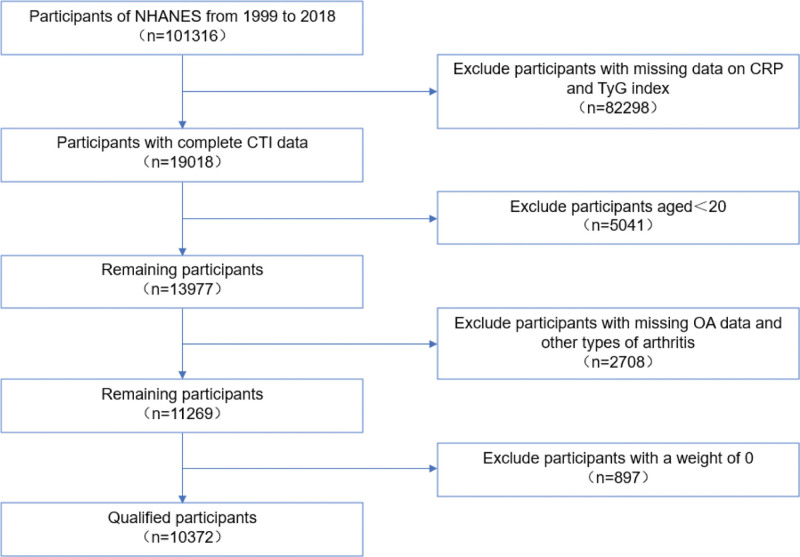
Participant flowchart of study. CTI = C-reactive protein-triglyceride glucose index, CRP = C-reactive protein, NHANES = National Health and Nutrition Examination Survey, OA = osteoarthritis.

We selected 1,01,316 participants from the NHANES data spanning 1999 to 2018. Participants with missing data for key variables – CRP, fasting triglycerides, and fasting blood glucose (FBG) – or those without OA diagnosis data were excluded. Additionally, individuals with other types of arthritis, those with a weight of zero, and those under 20 years of age were excluded. Multiple imputations were used to handle missing covariate data. Ultimately, data from 10,372 eligible participants with complete baseline information were used to investigate the correlation between the CTI and OA prevalence (Fig. 1). The NHANES ID codes used for data extraction are provided in Supplementary Material 1, Supplemental Digital Content, https://links.lww.com/MD/R666. Subsequently, we conducted further analysis of the OA cohort, excluding 2 participants with missing survival data. Ultimately, data from 1064 participants were included in the analysis to assess the role of CTI in stratifying mortality risk among patients with OA.

### 2.2. Assessment of OA

The NHANES surveys and collects data on OA status primarily based on the “Medical Conditions” questionnaire from the National Health Interview Survey. When participants were asked, “Has a doctor or other health professional ever told you that you had arthritis?” Participants who answered “Yes” to this question will be asked the next question: “What type of arthritis?” Participants diagnosed with “osteoarthritis or degenerative arthritis” were included in this study, whereas those with rheumatoid arthritis, psoriatic arthritis, and other types of arthritis were excluded. This process divided the study subjects into OA and non-OA groups. A self-reported physician’s diagnosis of OA is the primary case definition used in epidemiological studies.^[23]^ Previous studies have demonstrated that the concordance between self-reported and clinically confirmed OA is reliable.^[24]^

### 2.3. Assessment of CTI

CTI is calculated using the following formula^[12,25]^:


$$\begin{array}{l} \begin{array}{lll} & TyG=ln[(fasting\;triglycerides\left(TG\right)\left(mg/dL\right)\; & \times fasting\;blood\;glucose\left(FBG\right)\left(mg/dL\right)/\;2]\; \end{array} \end{array}$$

$$\begin{array}{l} CTI^{}=0.412\times ln\left(CRP\right)+TyG. \end{array}$$

### 2.4. Covariates

This study incorporated a series of covariates that may influence OA, based on clinical considerations and prior research, and evaluated them in subsequent analyses. These include sociodemographic characteristics such as age, gender, ethnicity, education, smoking habits, drinking status, poverty income ratio, body mass index (BMI), physical activity level, and laboratory indicators (CRP, FBG, triglycerides, high-density lipoprotein, and low-density lipoprotein). Clinical conditions include cardiovascular disease, hypertension, diabetes, and pharmacotherapy treatments (such as glucocorticoids, antihyperlipidemic, antihyperglycemic, and antihypertensive drugs).

These data were derived from standardized NHANES questionnaires, laboratory tests, and physical examinations. Demographic variables such as age, gender, and ethnicity were collected via questionnaire, with ethnicity categorized as Mexican American, non-Hispanic Black, non-Hispanic White, other Hispanic, other race. Educational levels were classified into 3 categories: less than high school, high school or equivalent, college or above. Smoking habits were defined as never, former, or now. Drinking status were categorized based on lifetime drinking history, drinking frequency over the past 12 months, and average daily consumption on drinking days: never, former, mild, moderate, heavy. The BMI of all participants was recorded by examiners at the examination center and calculated as weight in kilograms divided by height in meters squared (kg/m^2^). Physical activity levels were assessed using the NHANES Physical Activity Questionnaire. Metabolic equivalent values were calculated based on standardized energy expenditure. Subsequently, physical activity levels were categorized as insufficient or sufficient according to the Physical Activity Guidelines for Americans, using 600 MET-min/wk as the cutoff point. Clinical history was collected through standardized medical questionnaires and examination data: cardiovascular diseases included self-reported physician-diagnosed congestive heart failure, coronary artery disease, angina pectoris, or myocardial infarction; hypertension was defined as systolic blood pressure ≥140 mm Hg, diastolic blood pressure ≥90 mm Hg, prior hypertension diagnosis, or current use of antihypertensive medications. Diabetes was defined as having a prior diagnosis, currently taking antihyperglycemic or insulin, FBG ≥ 126 mg/dL, 2-hour postprandial blood glucose ≥200 mg/dL, or glycated hemoglobin ≥6.5%. Pharmacotherapy treatments were assessed through medication questionnaires and medication bottle inspections. Participants were asked whether they had used any medications in the past 30 days and categorized as “yes” or “no” based on their response. If “yes” was reported, they were required to list the specific medications used.

### 2.5. Statistical analysis

Given that NHANES employs a complex multistage sampling design, we weighted the survey sample data to obtain nationally representative results. All analyses presented below were based on weighted data. For continuous variables with a normal or approximately normal distribution, data are represented by the mean and standard deviation. For continuously distributed variables with skewed distributions, data are represented by the median and interquartile range. Categorical variables are described as frequency and percentage (%). Comparisons between groups for categorical variables were performed using chi-square tests, while continuous variables were analyzed using Student’s t tests or 1-way analysis of variance. Multiple-factor logistic regression analysis was employed to adjust for confounding factors and assess the association between the CTI and OA prevalence. The corresponding results are expressed as odds ratios (OR) with 95% confidence intervals (95% CI). In the logistic regression model, model 1 was the unadjusted model; model 2 was adjusted for age, gender, and ethnicity; model 3 was a fully adjusted model that further adjusted for education, smoking habits, drinking status, poverty income ratio, BMI, hypertension, diabetes, high-density lipoprotein, low-density lipoprotein, cardiovascular disease, physical activity level, taking antihyperlipidemic, antihyperglycemic, antihypertensive, and glucocorticoids based on model 2. Convert the CTI from a continuous variable to a categorical variable using quartiles (Q1–Q4), with Q1 as the reference category, and conduct trend tests based on the CTI quartiles. Under the fully adjusted model, restricted cubic spline (RCS) analysis was constructed to depict the nonlinear relationship between CTI and OA prevalence. Compare the predictive capabilities of CRP, TyG index, and CTI for the prevalence of OA using receiver operating characteristic (ROC) analysis and the area under the curve (AUC). Additionally, we conducted Kaplan–Meier survival analysis and constructed Cox proportional hazards regression models to assess survival differences in all-cause mortality risk among OA populations across the CTI quartiles. The covariates adjusted in the 3 risk regression models were identical to those considered in the above-mentioned multiple logistic regression analysis model. Similarly, we employed RCS in our fully adjusted model to reveal a potential linear association between the CTI and the risk of all-cause mortality from OA. To ensure the reliability of the results, we conducted subgroup analyses to explore the relationship between CTI and OA within different subgroups. All data analyses were ultimately performed using R version 4.4.2. The R code used for statistical analysis can be found in Supplementary Material 2, Supplemental Digital Content, https://links.lww.com/MD/R666. A *P*-value < .05 (2-sided) indicates a statistically significant difference.

## 3. Results

### 3.1. Baseline characteristics of the study population

The cross-sectional component of this study examined the association between the CTI and OA prevalence. A total of 10,372 participants were included, with a weighted total population of 10,25,53,328. The average age was 44.28 years, and the female proportion was 50.64%. The mean CTI score was 7.96, and the prevalence of OA was 10.28%. The sociodemographic characteristics are shown in Table 1. Compared to participants without OA, those with OA were older, mostly female, had higher BMI, were more likely to be non-Hispanic white, and were more inclined to smoke and drink alcohol. Additionally, the OA group exhibited a significantly higher prevalence of comorbidities such as diabetes, hypertension, and cardiovascular disease. The longitudinal component of this study examined the relationship between CTI and all-cause mortality risk in patients with OA. A total of 1064 participants were included, with a mean age of 61.70 years, a mean CTI of 8.27, and 65.80% being female. The demographic characteristics of the participants are shown in Table 2. During the follow-up period, a total of 411 participants experienced death. Compared to survivors, non-survivors are typically older, more likely to drink alcohol, and tend to have lower levels of education and income. They also exhibit higher prevalence rates of diabetes, hypertension, and cardiovascular disease, often have a history of glucocorticoid use, and engage in less physical activity.

**Table 1:** Comparison of baseline characteristics between all participants with and without prevalent osteoarthritis in the cross-sectional analysis.

Characteristics	Overall (N = 10372)	Without OA (N = 9306)	With OA (N = 1066)	*P*-value
CTI	7.96 ± 0.02	7.89 ± 0.02	8.27 ± 0.03	<.001
Age	44.28 ± 0.30	42.33 ± 0.29	61.70 ± 0.43	<.001
PIR	3.06 ± 0.03	3.05 ± 0.03	3.14 ± 0.07	.18
FBG	101.43 ± 0.34	100.87 ± 0.36	106.50 ± 0.98	<.001
TG	137.89 ± 1.72	136.5 ± 1.83	149.99 ± 3.89	.002
TyG	8.64 ± 0.01	8.62 ± 0.01	8.82 ± 0.02	<.001
HDL	53.27 ± 0.25	52.99 ± 0.26	55.79 ± 0.62	<.001
LDL	118.09 ± 0.47	117.88 ± 0.50	120.00 ± 1.16	.08
BMI	28.08 ± 0.09	27.90 ± 0.09	29.73 ± 0.27	<.001
CRP	0.39 ± 0.01	0.38 ± 0.01	0.54 ± 0.03	<.001
Age				<.001
<65	8272 (86.71)	7772 (90.03)	500 (57.02)	
≥65	2100 (13.29)	1534 (9.97)	566 (42.98)	
Gender				<.001
Female	5299 (50.64)	4608 (48.95)	691 (65.74)	
Male	5073 (49.36)	4698 (51.05)	375 (34.26)	
Ethnicity				<.001
Mexican American	2297 (8.55)	2202 (9.30)	95 (1.83)	
Non-Hispanic Black	1844 (10.72)	1716 (11.27)	128 (5.81)	
Non-Hispanic White	5055 (69.91)	4286 (68.04)	769 (86.65)	
Other Hispanic	736 (5.20)	689 (5.57)	47 (1.86)	
Other race	440 (5.62)	413 (5.82)	27 (3.85)	
Education				.68
College or above	5081 (58.04)	4523 (58.19)	558 (56.76)	
High school or equivalent	2383 (24.25)	2135 (24.14)	248 (25.25)	
Less than high school	2908 (17.71)	2648 (17.67)	260 (17.99)	
Smoking habits				<.001
Former	2545 (24.06)	2164 (22.73)	381 (35.91)	
Never	5687 (53.49)	5167 (54.21)	520 (47.02)	
Now	2140 (22.45)	1975 (23.06)	165 (17.07)	
Drinking status				<.001
Former	1862 (14.89)	1585 (13.98)	277 (23.00)	
Heavy	2223 (21.84)	2150 (23.48)	73 (7.16)	
Mild	3438 (36.54)	2973 (35.36)	465 (47.08)	
Moderate	1390 (15.57)	1288 (16.12)	102 (10.65)	
Never	1459 (11.16)	1310 (11.06)	149 (12.11)	
BMI				<.001
<25	3267 (34.98)	3030 (36.05)	237 (25.42)	
≥25	7105 (65.02)	6276 (63.95)	829 (74.58)	
Hypertension				<.001
No	6660 (68.72)	6283 (71.86)	377 (40.67)	
Yes	3712 (31.28)	3023 (28.14)	689 (59.33)	
Diabetes				<.001
No	8980 (89.80)	8153 (90.80)	827 (80.87)	
Yes	1392 (10.20)	1153 (9.20)	239 (19.13)	
Taking antihyperlipidemic				<.001
No	9071 (88.61)	8329 (90.56)	742 (71.20)	
Yes	1301 (11.39)	977 (9.44)	324 (28.80)	
Taking antihyperglycemic				<.001
No	9693 (95.11)	8756 (95.68)	937 (90.03)	
Yes	679 (4.89)	550 (4.32)	129 (9.97)	
Taking antihypertensive				<.001
No	7902 (79.58)	7448 (83.06)	454 (48.52)	
Yes	2470 (20.42)	1858 (16.94)	612 (51.48)	
Cardiovascular disease				<.001
No	9543 (93.86)	8694 (95.05)	849 (83.25)	
Yes	829 (6.14)	612 (4.95)	217 (16.75)	
Taking glucocorticoids				<.001
No	10,237 (98.61)	9203 (98.77)	1034 (97.15)	
Yes	135 (1.39)	103 (1.23)	32 (2.85)	
Physical activity level				.11
<600 MET-min/wk	4950 (48.46)	4475 (48.79)	475 (45.50)	
≥600 MET-min/wk	5422 (51.54)	4831 (51.21)	591 (54.50)	

BMI = body mass index, CRP = C-reactive protein, CTI = C-reactive protein-triglyceride-glucose index, FBG = Fasting blood glucose, HDL = high density lipoprotein, LDL = low density lipoprotein, MET = Metabolic equivalent, PIR = poverty income ratio, TG = triglyceride, TyG = triglyceride-glucose.

**Table 2:** Comparison of baseline characteristics among osteoarthritis population based on survivor and non-survivor in the longitudinal study.

Characteristics	Total (N = 1064)	Survivor (N = 653)	Non-Survivor (N = 411)	*P*-value
CTI	8.27 ± 0.03	8.18 ± 0.04	8.46 ± 0.04	<.001
Age	61.70 ± 0.43	57.40 ± 0.48	71.09 ± 0.70	<.001
PIR	3.14 ± 0.07	3.37 ± 0.08	2.65 ± 0.08	<.001
FBG	106.50 ± 0.98	104.49 ± 0.97	110.88 ± 1.95	.003
TG	149.98 ± 3.89	145.79 ± 5.04	159.12 ± 5.42	.07
TyG	8.82 ± 0.02	8.78 ± 0.03	8.91 ± 0.03	.002
HDL	55.82 ± 0.62	55.54 ± 0.78	56.42 ± 1.07	.51
LDL	120.09 ± 1.16	121.97 ± 1.50	115.97 ± 2.24	.04
BMI	29.73 ± 0.27	30.13 ± 0.37	28.85 ± 0.38	.02
CRP	0.54 ± 0.03	0.46 ± 0.04	0.70 ± 0.08	.02
Gender				.58
Female	690 (65.80)	434 (66.35)	256 (64.60)	
Male	374 (34.20)	219 (33.65)	155 (35.40)	
Ethnicity				.10
Mexican American	95 (1.83)	64 (1.95)	31 (1.58)	
Non-Hispanic Black	127 (5.76)	90 (6.28)	37 (4.62)	
Non-Hispanic White	769 (86.80)	443 (85.14)	326 (90.41)	
Other Hispanic	47 (1.86)	33 (1.88)	14 (1.84)	
Other race	26 (3.75)	23 (4.75)	3 (1.56)	
Education				<.001
College or above	557 (56.80)	367 (60.29)	190 (49.20)	
High school or equivalent	247 (25.18)	162 (25.94)	85 (23.51)	
Less than high school	260 (18.02)	124 (13.77)	136 (27.29)	
Smoking habits				.22
Former	379 (35.80)	210 (34.17)	169 (39.35)	
Never	520 (47.10)	338 (49.17)	182 (42.60)	
Now	165 (17.10)	105 (16.66)	60 (18.05)	
Drinking status				.002
Former	282 (23.54)	149 (19.88)	133 (31.53)	
Heavy	72 (7.12)	51 (7.74)	21 (5.76)	
Mild	461 (46.74)	291 (48.94)	170 (41.95)	
Moderate	105 (10.83)	76 (12.45)	29 (7.29)	
Never	144 (11.76)	86 (10.98)	58 (13.47)	
Hypertension				<.001
No	377 (40.74)	267 (45.73)	110 (29.84)	
Yes	687 (59.26)	386 (54.27)	301 (70.16)	
Diabetes				<.001
No	826 (80.89)	532 (84.17)	294 (73.72)	
Yes	238 (19.11)	121 (15.83)	117 (26.28)	
Taking antihyperlipidemic				.23
No	740 (71.15)	464 (72.43)	276 (68.38)	
Yes	324 (28.85)	189 (27.57)	135 (31.62)	
Taking antihyperglycemic				<.001
No	935 (90.02)	593 (92.48)	342 (84.64)	
Yes	129 (9.98)	60 (7.52)	69 (15.36)	
Taking antihypertensive				<.001
No	453 (48.49)	317 (53.84)	136 (36.81)	
Yes	611 (51.51)	336 (46.16)	275 (63.19)	
Cardiovascular disease				<.001
No	847 (83.22)	560 (89.48)	287 (69.57)	
Yes	217 (16.78)	93 (10.52)	124 (30.43)	
Taking glucocorticoids				.005
No	1032 (97.15)	642 (98.46)	390 (94.29)	
Yes	32 (2.85)	11 (1.54)	21 (5.71)	
Physical activity level				<.001
<600 MET-min/wk	459 (44.69)	230 (39.42)	229 (56.18)	
≥600 MET-min/wk	605 (55.31)	423 (60.58)	182 (43.82)	

BMI = body mass index, CRP = C-reactive protein, CTI = C-reactive protein-triglyceride-glucose index, FBG = Fasting blood glucose, HDL = high density lipoprotein, LDL = low density lipoprotein, MET = Metabolic equivalent, PIR = poverty income ratio, TG = triglyceride, TyG = triglyceride-glucose.

### 3.2. Association between CTI and OA

**Figure 2.**
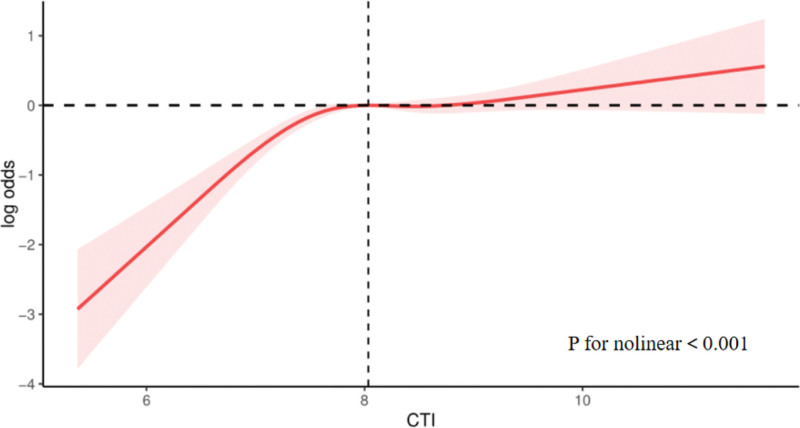
Restricted cubic spline analysis of the association between C-reactive protein-triglyceride glucose index and osteoarthritis prevalence. CTI = C-reactive protein-triglyceride glucose index.

The multi-factor logistic regression analysis shown in Table 3 indicates that when CTI was treated as a continuous variable, all models demonstrated a positive correlation between the CTI and the prevalence of OA. The fully adjusted Model yielded an OR of 1.35 (95% CI: 1.21–1.50). When the CTI was categorized into quartiles, a significant positive trend was observed (*P* for trend <.001). In model 3, the prevalence of OA among participants in the highest CTI quartile (Q4) was 1.37 times (OR = 2.37, 95% CI: 1.75–3.22) higher than that among those in the lowest quartile (Q1). Furthermore, RCS analysis revealed a nonlinear relationship between the CTI and OA prevalence, as shown in Figure 2.

**Table 3:** Associations between C-reactive protein-triglyceride-glucose index and osteoarthritis.

Exposure	Model 1		Model 2		Model 3	
	OR (95% CI)	*P*-value	OR (95% CI)	*P*-value	OR (95% CI)	*P*-value
CTI	1.55 (1.44, 1.67)	<.001	1.46 (1.35, 1.59)	<.001	1.35 (1.21, 1.50)	<.001
CTI quartile						
Q1 (4.76, 7.39)	ref		ref		ref	
Q2 (7.39, 8.03)	2.69 (2.03, 3.57)	<.001	2.49 (1.87, 3.31)	<.001	2.28 (1.71, 3.05)	<.001
Q3 (8.03, 8.64)	3.28 (2.45, 4.39)	<.001	2.73 (2.01, 3.69)	<.001	2.29 (1.67, 3.14)	<.001
Q4 (8.64, 13.30)	3.75 (2.92, 4.80)	<.001	3.06 (2.34, 3.99)	<.001	2.37 (1.75, 3.22)	<.001
*P* for trend		<.001		<.001		<.001

Model 1 non-adjusted.

Model 2 adjusted for age, gender, and ethnicity.

Model 3 was further adjusted for education, smoking habits, drinking status, PIR, hypertension, diabetes, BMI, HDL, LDL, Cardiovascular disease, physical activity level, antihyperlipidemic, antihyperglycemic, antihypertensive, and glucocorticoid use.

BMI = body mass index, CTI = C-reactive protein-triglyceride-glucose index, HDL = high density lipoprotein, LDL = low density lipoprotein, OR = odds ratios, PIR = poverty income ratio.

### 3.3. Subgroup analysis of the association between CTI and OA

Subgroup analysis in Table 4 confirmed the robustness of the positive association between CTI and OA prevalence across most demographic and clinical strata, including age, gender, smoking habits, or history of hypertension. Interestingly, we observed that the correlation between CTI and OA was more pronounced in individuals with BMI < 25. The reason may be that these populations inherently possess a higher risk of developing OA, leading to an increased likelihood of adverse outcomes. This highlights the potential significance of CTI within the subgroup.

**Table 4:** Subgroup analysis of the association between C-reactive protein-triglyceride glucose index and osteoarthritis.

Variables	95% CI	*P*-value	*P* for interaction
Age			.13
<65	1.30 (1.14, 1.48)	<.001	
≥65	1.22 (1.01, 1.47)	.04	
Gender			.52
Male	1.37 (1.13, 1.66)	.002	
Female	1.36 (1.18, 1.58)	<.001	
Ethnicity			.85
Non-Hispanic White	1.39 (1.23, 1.57)	<.001	
Non-Hispanic Black	1.13 (0.87, 1.47)	.37	
Mexican American	1.31 (0.95, 1.82)	.10	
Other Hispanic	0.95 (0.54, 1.67)	.86	
Other Race	1.01 (0.65, 1.58)	.96	
Smoking habits			.65
Former	1.32 (1.09, 1.60)	.01	
Now	1.37 (1.06, 1.76)	.02	
Never	1.38 (1.16, 1.64)	<.001	
Drinking status			.85
Moderate	1.38 (0.97, 1.96)	.07	
Never	1.29 (0.94, 1.78)	.11	
Mild	1.31 (1.11, 1.55)	.002	
Heavy	1.44 (0.86, 2.44)	.17	
Former	1.33 (1.07, 1.67)	.01	
Hypertension			.21
Yes	1.24 (1.08, 1.43)	.004	
No	1.39 (1.16, 1.66)	<.001	
Diabetes			.004
No	1.47 (1.31, 1.65)	<.001	
Yes	0.91 (0.72, 1.15)	.42	
BMI			<.001
≥25	1.20 (1.06, 1.36)	.005	
<25	1.82 (1.44, 2.30)	<.001	
Taking glucocorticoids			.19
No	1.34 (1.20, 1.50)	<.001	
Yes	2.43 (0.76, 7.79)	.13	

BMI = body mass index.

### 3.4. ROC analysis of CRP, TyG index and CTI to OA

**Figure 3.**
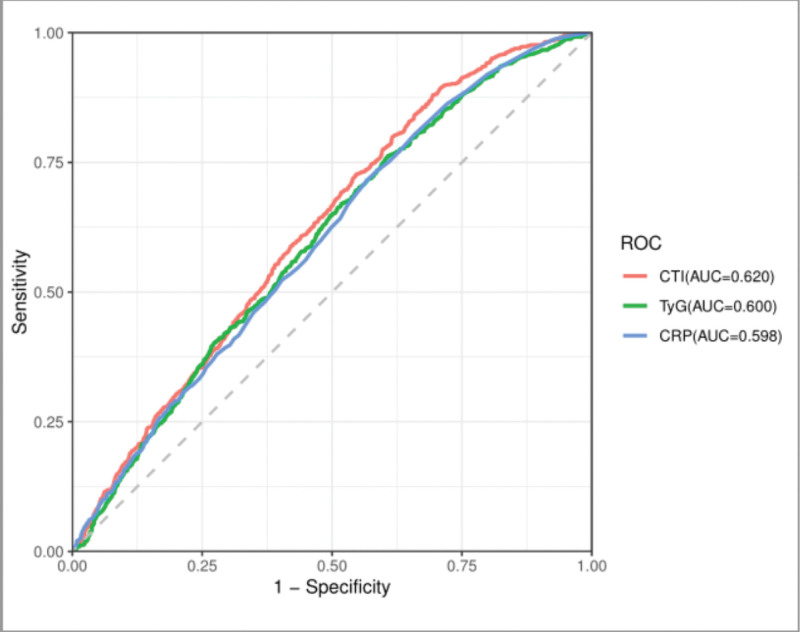
Comparing the discriminatory value of C-reactive protein, triglyceride-glucose index, and C-reactive protein-triglyceride glucose index for osteoarthritis using receiver operating characteristic curves. CRP = C-reactive protein, CTI = C-reactive protein-triglyceride glucose index, ROC = receiver operating characteristic, TyG = triglyceride-glucose.

To evaluate the predictive ability of different biomarkers for OA, we compared the ROC curves of CTI, CRP, and TyG index. Notably, the CTI demonstrated superior discriminatory performance with an AUC of 0.620 (Fig. 3). This value exceeded the results of CRP (AUC = 0.598) and TyG index (AUC = 0.600) when measured individually, indicating that the composite CTI provides more precise diagnostic outcomes for OA compared to its individual components.

### 3.5. Association between CTI and all-cause mortality risk in the OA population

**Figure 4.**
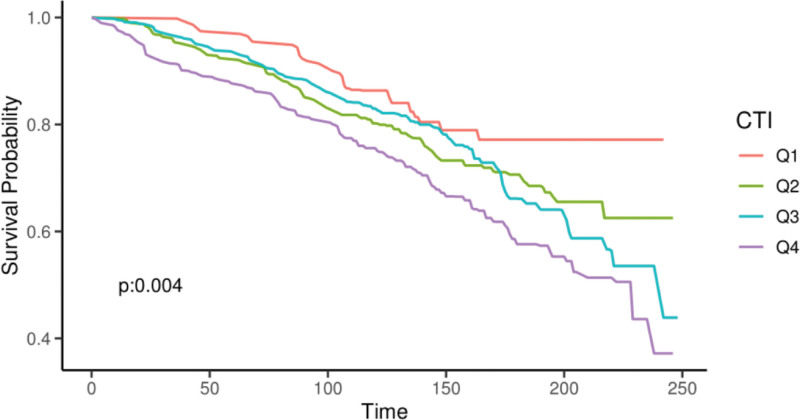
Kaplan–Meier survival curves according to C-reactive protein-triglyceride glucose index quartiles investigating the relationship between C-reactive protein-triglyceride glucose index and all-cause mortality outcomes in the osteoarthritis cohort. CTI = C-reactive protein-triglyceride glucose index.

**Figure 5.**
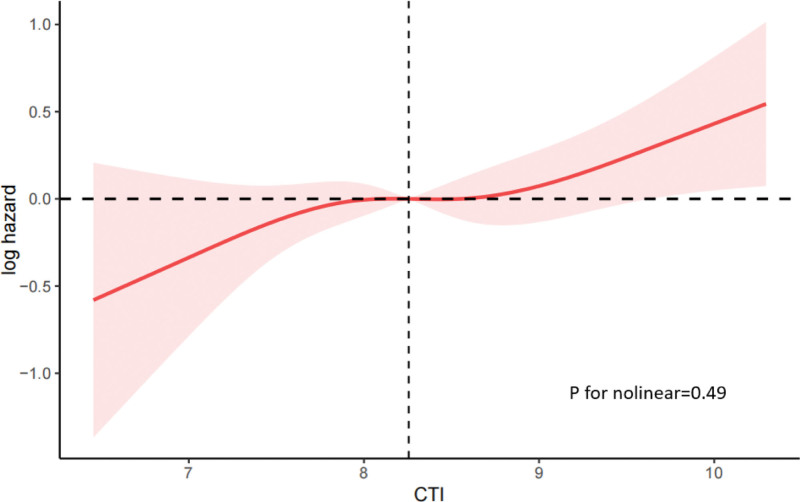
Restricted cubic spline analysis of the association between C-reactive protein-triglyceride glucose index and all-cause mortality in the osteoarthritis population. CTI = C-reactive protein-triglyceride glucose index.

We further explored the prognostic value of the CTI in patients with OA. The Cox proportional hazards model in Table 5 demonstrates that elevated CTI values were significantly associated with an increased all-cause mortality risk. After full adjustment, each unit increase in the CTI corresponded to a 24% increase in mortality risk (HR = 1.24, 95% CI: 1.03–1.50, *P* = .02). Patients in the highest quartile (Q4) of the CTI had a 62% higher mortality risk compared to those in the lowest quartile (HR = 1.62, 95% CI: 1.07–2.45, *P* = .02). The Kaplan–Meier survival curve (Fig. 4) visually confirmed this graded association, showing a significant decline in cumulative survival probability with increasing CTI quartiles (*P* = .004). RCS analysis further demonstrated a linear relationship between CTI and mortality risk in the OA cohort (Fig. 5).

**Table 5:** Associations between C-reactive protein-triglyceride glucose index and all-cause mortality outcomes in the osteoarthritis population.

Exposure	Model 1		Model 2		Model 3	
	HR (95% CI)	*P*-value	HR (95% CI)	*P*-value	HR (95% CI)	*P*-value
CTI	1.38 (1.20, 1.59)	<.001	1.30 (1.14, 1.49)	<.001	1.24 (1.03, 1.50)	.02
CTI quartile						
Q1 (4.76, 7.39)	ref		ref		ref	
Q2 (7.39, 8.03)	1.41 (0.88, 2.25)	.15	1.38 (0.97, 1.96)	.07	1.42 (0.99, 2.03)	.06
Q3 (8.03, 8.64)	1.48 (0.93, 2.35)	.10	1.26 (0.86, 1.84)	.23	1.29 (0.86, 1.94)	.22
Q4 (8.64, 13.30)	2.05 (1.35, 3.11)	<.001	1.76 (1.25, 2.46)	.001	1.62 (1.07, 2.45)	.02
*P* for trend		<.001		.002		.09

Model 1 non-adjusted.

Model 2 adjusted for age, gender, and ethnicity.

Model 3 further adjusted for education, smoking habits, drinking status, PIR, hypertension, diabetes, BMI, HDL, LDL, cardiovascular disease, physical activity level, taking antihyperlipidemic, taking antihyperglycemic, taking antihypertensive, and taking glucocorticoids.

BMI = body mass index, CTI = C-reactive protein-triglyceride-glucose index, HDL = high density lipoprotein, LDL = low density lipoprotein, OR = odds ratios, PIR = poverty income ratio.

### 3.6. Subgroup analysis of the association between CTI and all-cause mortality risk in the OA population

We further examined the association between the CTI and all-cause mortality risk in the OA cohort across different classes through a subgroup analysis (Table 6). In nearly all subgroups, an elevated CTI showed a positive correlation with increased all-cause mortality risk, with no significant interactions observed. This indicates that the observed positive correlation remained unaffected by these variables.

**Table 6:** Subgroup analysis of the association between C-reactive protein-triglyceride glucose index and all-cause mortality in the osteoarthritis population.

Variables	95% CI	*P*-value	*P* for interaction
Age			.25
<65	2.27 (1.38, 3.70)	.002	
≥65	1.39 (1.01, 1.91)	.04	
BMI			.04
<25	2.99 (1.44, 6.22)	.01	
≥25	1.35 (1.02, 1.80)	.04	
Education			.33
College or above	2.06 (1.38, 3.07)	<.001	
Less than high school	1.57 (0.85, 2.89)	.14	
High school or equivalent	1.04 (0.56, 1.91)	.91	
Hypertension			.002
Yes	1.35 (0.97, 1.88)	.08	
No	2.48 (1.64, 3.76)	<.001	
Diabetes			.73
No	1.55 (1.11, 2.18)	.01	
Yes	2.24 (1.36, 3.68)	.004	
Taking glucocorticoids			.01
No	1.73 (1.31, 2.28)	<.001	
Yes	2.59 (0.17, 38.91)	.38	
Physical activity level			.29
<600 MET-min/wk	1.28 (0.84, 1.94)	.24	
≥600 MET-min/wk	2.11 (1.36, 3.29)	.001	

BMI = body mass index, MET = metabolic equivalent.

## 4. Discussion

To our knowledge, this is the first large-scale study to propose the CTI as a diagnostic biomarker for OA. As a composite index integrating inflammatory and metabolic factors, its diagnostic performance surpasses that of individual markers such as CRP or the TyG index. Additionally, we observed a significant positive linear correlation between elevated CTI levels and all-cause mortality risk in the OA cohort. This indicates that higher CTI scores are associated with increased all-cause mortality risk among individuals with OA. These associations remained consistent across most subgroups. Notably, the estimated prevalence of OA showed a comparable magnitude of effect between participants with and without overweight, those with and without hypertension complications, and younger and older individuals. These findings suggest that individuals with lower CTI levels have a lower prevalence and better prognosis for OA.

Previous studies have separately analyzed the independent effects of CRP and the TyG index on OA, consistent with our findings. A large-scale prospective cohort study from the UK Biobank indicated that elevated CRP levels were associated with an increased risk of OA.^[26]^ In individuals with metabolic impairment, elevated CRP levels significantly increased the risk of developing OA by 35%. Additionally, Canadian researchers Perruccio et al^[27]^ have proposed that a dose-response relationship may exist between CRP levels and OA symptoms. Meanwhile, a recent analysis of the Korean NHANES once again revealed a significant positive association between the TyG index and knee OA.^[28]^ Moreover, several studies have demonstrated that CRP and insulin resistance are closely associated with poor disease outcomes and mortality.^[10,29]^ Therefore, exploring the association between a novel biomarker that integrates both factors and OA represents a key advancement in understanding the relationship between the metabolic and inflammatory co-state and OA.

CTI can be more accurately linked to OA and its adverse outcomes, potentially due to its ability to concurrently capture 2 key pathophysiological processes: systemic inflammation and insulin resistance.^[30]^ Firstly, chronic low-grade inflammation driven by the innate immune system is a core component in the pathogenesis of OA.^[31,32]^ It is widely accepted that CRP serves as an effective biomarker that reflects systemic inflammation levels.^[33]^ Elevated CRP levels promote the onset and progression of OA by increasing cartilage degeneration and osteophyte formation through mediating the activity and differentiation of macrophages and osteoclasts.^[34,35]^ Secondly, insulin serves as a signaling factor regulating synovial inflammation and catabolic processes. Its resistance impairs the body’s ability to suppress inflammatory mediators, thereby increasing the risk of OA.^[36]^ At the same time, insulin resistance prompts the pancreas to secrete more insulin, which increases lipid synthesis and storage, leading to obesity. Obesity not only directly affects the progression of OA through excessive mechanical loading on cartilage tissue but also by inducing inflammation associated with adipose tissue.^[37]^ Moreover, fat deposition leads to elevated plasma levels of TNF-α and IL-6, with TNF being a key factor causing insulin resistance in both adipose and non-adipose tissues.^[38,39]^ Therefore, a vicious cycle between insulin resistance and obesity may promote OA development. Meanwhile, a series of studies have revealed a strong association between chronic inflammation, insulin resistance, and overall mortality, though the underlying pathophysiological mechanisms are highly complex. This association may be linked to the aging process, which involves multiple cell types, signaling pathways, and molecular processes.^[40-42]^ Persistent inflammatory responses will impair immune defense functions, leading to tissue and organ damage.^[43,44]^ Under high insulin levels, chondrocytes exhibit impaired autophagy.^[45]^ Chronic hyperglycemia exacerbates oxidative stress, amplifying the functional effects of pro-inflammatory cytokines and advanced glycation end products, thereby diminishing the chondrogenic differentiation potential of stem cells, impeding cartilage repair, and accelerating chondrocyte apoptosis.^[46]^ Finally, recent studies have once again confirmed the interactive relationship between inflammatory pathways and insulin resistance. On 1 hand, pro-inflammatory cytokines can disrupt insulin signaling by inhibiting the phosphorylation of insulin receptor substrate-1.^[47]^ On the other hand, insulin resistance can also induce mitochondrial dysfunction, thereby triggering metabolic disorders and exacerbating oxidative stress, which further promotes inflammatory responses, forming a vicious cycle.^[48]^ CTI is a simple, readily accessible, and novel biomarker that can be calculated from routine laboratory parameters, thereby avoiding additional financial burden for patients. It offers a complementary pathophysiological perspective on the metabolic and inflammatory states, demonstrating promising application prospects.

In summary, this study had several commendable aspects. This was a nationwide representative cohort study. Its large sample size provides reliable statistical power, offering new insights into assessing the risk of OA and all-cause mortality. At the meantime, we adjusted for potential confounding factors that could have influenced the results to ensure the greater reliability of this study’s findings. Moreover, we conducted subgroup analyses to investigate the robustness of the association between the CTI and OA across different populations.

However, despite these strengths, certain limitations of this study should be noted. Firstly, as an observational study, it is inconclusive to establish a causal relationship between the CTI and OA. Secondly, despite accounting for multiple confounding factors, it may still be difficult to eliminate the influence of all potential factors. Third, as a self-reported database, NHANES may be subject to recall bias. Additionally, self-reported questionnaires cannot determine the radiographic staging of OA. Finally, the NHANES primarily represents the US population, and other regions and ethnic groups remain worthy of further investigation.

## 5. Conclusion

In this large-scale, nationally representative cohort study, we demonstrate for the first time that the CTI is significantly and independently associated with both the prevalence of OA and all-cause mortality in patients with OA. CTI showed stronger associations with OA risk compared to traditional biomarkers such as CRP and TyG index, suggesting its potential value as a composite indicator reflecting both inflammatory and metabolic pathways. These findings indicate that CTI, as a readily accessible noninvasive biomarker, may be associated with OA risk and prognosis. If future prospective or interventional studies confirm its predictive value, CTI assessment might aid in early risk stratification and inform personalized intervention strategies, particularly in high-risk populations. Further prospective intervention studies are needed to validate its causal role and evaluate its application value in guiding OA prevention and treatment.

## Acknowledgments

Thanks to all NHANES participants for their contributions.

## Author contributions

**Conceptualization:** Rong Xie.

**Formal analysis:** Rong Xie.

**Investigation:** Juncheng Long.

**Methodology:** Rong Xie.

**Supervision:** Dongguang Liu.

**Validation:** Juncheng Long.

**Writing – original draft:** Rong Xie.

**Writing – review & editing:** Dongguang Liu.

## Supplementary Material


