# Supplementary material for: C-reactive protein-triglyceride glucose index is a reliable biomarker for osteoarthritis: A cross-sectional study based on NHANES 1999–2018
Source: Medicine (Baltimore). 2026 Apr 17;105(16):e48314. doi: 10.1097/MD.0000000000048314 (PMC13095320; doi:10.1097/MD.0000000000048314)
Supplement: Supplementary file 1 [file medi-105-e48314-s001.pdf]

Supplementary Material 1. The NHANES ID codes used for data extraction.

	NHANES ID codes
Age	RIDAGEYR
PIR	INDFMPIR
FBG	LBXGLU
TG	LBXTR
HDL	LBDHDD, LBDHDL
LDL	LBDLDL
HBA1c	LBXGH
BMI	BMXBMI
CRP	LBXCRP, LB2CRP, LBDHRPLC, LBXHSCRP, LB2CRP
Gender	RIAGENDR
Ethnicity	RIDRETH1
Education	DMDEDUC2
Smoking habits	SMQ020, SMQ040
Drinking status	ALQ111, ALQ121, ALQ130, ALQ142, ALQ270, ALQ280, ALQ290, ALQ151 ALQ170
Hypertension	BPQ020
Diabetes	DIQ010
Taking antihyperlipidemic	Refer to Appendix 2: Standardized Generic Prescription Medication Names
Taking antihyperglycemic	
Taking glucocorticoids	
Taking antihypertensive	
Cardiovascular disease	MCQ160C, MCQ160B, MCQ160D,
Physical activity level	CVXPARC, PADMETS, PAQ605, PAQ635
Osteoarthritis definition	MCQ160A, MCQ190, MCQ191, MCQ195

Supplementary Material 2. The R code used for statistical analysis.

```
library(nhanesR)
setwd("C:\\Users\\xiaob\\Desktop\\CTI")

d1 <- dex_TyG(years = c(1999:2018))



d2 <- db_demo(years = c(1999:2018),ageyr = "Age",sex = "Sex",eth1 = "Race",
                    edu = "Education",
                    poverty = "PIR",Year = T) |>
    db_bodyMeasure(years = c(1999:2018),
                        BMI_kg.m2 = "BMI") |>
    db_HemalBiochemistry(years = c(1999:2018),fast_glucose_mg.dl = "FBG",
                             fast_triglyceride_mg.dl = "TG",
                             C_reactive_protein_mg.dl = "CRP",
                             hdl_cholesterol_mg.dl = "HDL_C",
                             ldl_cholesterol_mg.dl = "LDL_C",
                             wtsaf2yr = T,wtsaf4yr = T) |>
    diag_smoke(years = c(1999:2018)) |>
    diag_alcohol.user(years = c(1999:2018)) |>

    diag_Hypertension(years = c(1999:2018)) |>

    diag_DM(years = c(1999:2018))


d4 <- diag_arthritis(years = c(1999:2018),arthritis = T,arghritis_type = T,
                          osteoarthritis_or_degenerative_arthritis = T)



d0 <- Left_Join(d2,d1)

d0 <- Left_Join(d0,d4)

write.xlsx(x = d0,file = "d0.xlsx")
```

```
d0 <- drop_row(d0,is.na(d0$CRP))
d0 <- drop_row(d0,is.na(d0$TyG))
missValue(d0)
d0$CTI <- 0.412 * ifelse(d0$CRP > 0, log(d0$CRP), NA) + d0$TyG




write.xlsx(x = d0,file = "d8.xlsx")



d5 <- read.xlsx("d9.xlsx")


d3<-dex_PhysicalActivity(years = (1999:2018),all.5 = TRUE,
                                      MET=TRUE,total_MET=TRUE) |>
      drug_anti.Hyperlipidemic(years = c(1999:2018),
                                      take_drug = "Antihyperlipidemic") |>
      drug_anti.Diabetic(take_drug = "Antihyperglycemic")|>
      drug_anti.Hypertensive(take_drug = "Antihypertensive")
d3 <- d3 %>%
      mutate(PA_total_METQ = ifelse(PA_total_MET < 600, "<600",
                                      ifelse(PA_total_MET      >=      600      ,
"≥600",NA)))   #  处理缺失值
d3 <- drop_col(d3,"PA_total_MET")


d6 <- drug_search("glucocorticoids",years =c(1999:2018) )
d6 <- Drug("glucocorticoids",years = c(1999:2018) )




d5 <- Left_Join(d5,d3)

d5 <- Left_Join(d5,d6)

d6 <- d5 %>%
      mutate(nhs_wt = case_when(
            Year >= 1990 & Year <= 2000 ~ wtsaf4yr * 2 / 10,
            Year >= 2001 & Year <= 2002 ~ wtsaf4yr * 2 / 10,
            Year >= 2003 & Year <= 2004 ~ wtsaf2yr * 1 / 10,
            Year >= 2005 & Year <= 2006 ~ wtsaf2yr * 1 / 10,
            Year >= 2007 & Year <= 2008 ~ wtsaf2yr * 1 / 10,
```

```
              Year >= 2009 & Year <= 2010 ~ wtsaf2yr * 1 / 10,
              Year >= 2011 & Year <= 2012 ~ wtsaf2yr * 1 / 10,
              Year >= 2013 & Year <= 2014 ~ wtsaf2yr * 1 / 10,
              Year >= 2015 & Year <= 2016 ~ wtsaf2yr * 1 / 10,
              Year >= 2017 & Year <= 2018 ~ wtsaf2yr * 1 / 10))


d8 <- d6[d6$nhs_wt != 0, ]



d9 <- drop_col(d8,"wtsaf2yr","wtsaf4yr","arthritis","arghritis_type","Year",

                   "osteoarthritis_or_degenerative_arthritis")
summary(d9)


# Recode(d9$Race)
d9$Race <- Recode(d9$Race,
                      "Non-Hispanic White::",
                      "Non-Hispanic Black::",
                      "Mexican American::",
                      "Other Hispanic::",
                      "Other Race - Including Multi-Racial::Other Race",
                      to.numeric = FALSE)
# Recode(d9$Education)
d9$Education <- Recode(d9$Education,
                          "College Graduate or above::College or above",
                          "9-11th Grade (Includes 12th grade with no
diploma)::Less than high school",
                          "High School Grad/GED or Equivalent::High school or
equivalent",
                          "Some College or AA degree::College or above",
                          "Less Than 9th Grade::Less than high school",
                          "NA::",
                          to.numeric = FALSE)

# Recode(d9$DM)
d9$DM <- Recode(d9$DM,
                    "no::no",
                    "DM::yes",
                    "IFG::no",
                    "IGT::no",
                    "NA::",
```

```
                    to.numeric = FALSE)
# Recode(d9$Antihyperlipidemic)
d9$Antihyperlipidemic <- Recode(d9$Antihyperlipidemic,
                                "other::no",
                                "no::no",
                                "yes::yes",
                                "NA::",
                                to.numeric = FALSE)
# Recode(d9$Antihyperglycemic)
d9$Antihyperglycemic <- Recode(d9$Antihyperglycemic,
                               "other::no",
                               "no::no",
                               "yes::yes",
                               "NA::",
                               to.numeric = FALSE)
# Recode(d9$Antihypertensive)
d9$Antihypertensive <- Recode(d9$Antihypertensive,
                              "yes::yes",
                              "no::no",
                              "other::no",
                              "NA::",
                              to.numeric = FALSE)
# Recode(d9$take_drug)
d9$take_drug <- Recode(d9$take_drug,
                       "yes::yes",
                       "no::no",
                       "other::no",
                       "NA::",
                       to.numeric = FALSE)

summary(d9)

d9 <- d9 %>%
    mutate(BMIQ = ifelse(BMI < 25, "<25",
                         ifelse(BMI >= 25 , "≥25",NA)))   #  处理缺失值
d9 <- d9 %>%
    mutate(AgeQ = ifelse(Age < 65, "<65",
                         ifelse(Age >= 65 , "≥65",NA)))   #  处理缺失值
d9$CTIQ <- cut(
    d9$CTI,
    breaks = quantile(d9$CTI, probs = c(0, 0.25, 0.5, 0.75, 1)),
    labels = c("Q1", "Q2", "Q3", "Q4"),
    include.lowest = TRUE
)
```

```
quantile_values1 <- quantile(
        d9$CTI,
        probs = c(0, 0.25, 0.5, 0.75, 1)
)
print(quantile_values1)


d2 <- diag_CVD(years = c(1999:2018))
d9 <- Left_Join(d9,d2)


library(missRanger)
set.seed(123)
data.imp<- missRanger(d9)

write.xlsx(data.imp,file = "data.imp.xlsx")


library(survey)
nhs <- svy_design(data.imp)


svy_population(nhs)






svy_tableone(design = nhs,cv = c("CTI","Age","PIR", "FBG",
                                 "TG","TyG","HDL_C","LDL_C",
                                 "BMI","CRP"),
             c_meanPMse = T,
             gv = c("AgeQ","Sex","Race","Education","smoke",
                    "alcohol.user","BMIQ","Hypertension",
                    "DM","Antihyperlipidemic","Antihyperglycemic",
                    "Antihypertensive","CVD","take_drug","PA_total_METQ"),
             g_nSQper = T,round = 3,by = "OA",xlsx = "tableOA1.xlsx")
svy_tableone(design = nhs,cv = c("CTI","Age","PIR", "FBG",
                                 "TG","TyG","HDL_C","LDL_C",
                                 "BMI","CRP"),
             c_meanPMse = T,
```

```
                gv = c("AgeQ","Sex","Race","Education","smoke",
                        "alcohol.user","BMIQ","Hypertension",
                        "DM","Antihyperlipidemic","Antihyperglycemic",
                        "Antihypertensive","CVD","take_drug","PA_total_METQ"),
                g_nSQper = T,round = 3,xlsx = "tableOAall1.xlsx")
svy_tableone(design = nhs,cv = c("CTI","Age","PIR", "FBG",
                                       "TG","TyG","HDL_C","LDL_C",
                                       "BMI","CRP"),
                c_meanPMse = T,
                gv = c("AgeQ","Sex","Race","Education","smoke",
                        "alcohol.user","BMIQ","Hypertension",
                        "DM","OA","Antihyperlipidemic","Antihyperglycemic",
                        "Antihypertensive","CVD","take_drug","PA_total_METQ"),
                g_nSQper = T,round = 3,by = "CTIQ",xlsx = "tableCTIQ1.xlsx")
svy_tableone(design = nhs,cv = c("CTI","Age","PIR", "FBG",
                                       "TG","TyG","HDL_C","LDL_C",
                                       "BMI","CRP"),
                c_meanPMse = T,
                gv = c("AgeQ","Sex","Race","Education","smoke",
                        "alcohol.user","BMIQ","Hypertension",
                        "DM","OA","Antihyperlipidemic","Antihyperglycemic",
                        "Antihypertensive","CVD","take_drug","PA_total_METQ"),
                g_nSQper = T,round = 3,by = "CTIQ",xlsx = "tableCTIQall1.xlsx")



# 多因素
f0 <- svyglm(OA~CTI,nhs,family = quasibinomial)|> reg_table()
f1 <- svyglm(OA~CTI+AgeQ+Sex+Race,
                nhs,family = quasibinomial)|> reg_table()
f2 <- svyglm(OA~CTI+AgeQ+Sex+Race+Education+

smoke+alcohol.user+PIR+Hypertension+DM+BMIQ+HDL_C+LDL_C+
                    CVD+PA_total_METQ+take_drug+Antihyperlipidemic+
                    Antihyperglycemic+Antihypertensive,
                nhs,family = quasibinomial)|> reg_table()
crude.Model.n(f0,f1,f2,style = 1,xlsx = "CTI 多因素 1.xlsx")


f0 <- svyglm(OA~CTIQ,nhs,family = quasibinomial)|> reg_table()
f1 <- svyglm(OA~CTIQ+AgeQ+Sex+Race,
                nhs,family = quasibinomial)|> reg_table()
f2 <- svyglm(OA~CTIQ+AgeQ+Sex+Race+Education+
```

```
smoke+alcohol.user+PIR+Hypertension+DM+BMIQ+HDL_C+LDL_C+
                    CVD+PA_total_METQ+take_drug+Antihyperlipidemic+
                    Antihyperglycemic+Antihypertensive,
               nhs,family = quasibinomial)|> reg_table()
crude.Model.n(f0,f1,f2,style = 1,xlsx = "CTIQ 多因素 1.xlsx")




# RCS
library(rms)

f8 <- svyglm(OA~rcs(CTI,4)+AgeQ+Sex+Race+Education+

smoke+alcohol.user+PIR+Hypertension+DM+BMIQ+HDL_C+LDL_C+
                    CVD+Antihyperlipidemic+take_drug+PA_total_METQ+
                    Antihypertensive+Antihyperglycemic,
               design = nhs,family = quasibinomial)
optimal_nKnots(f8)
r8 <- RCS(f8)
ggplot(r8)
getChangepoints(r8)






stratum_model(object= nhs,
                y = "OA",
                x = "CTI",
                stratum = "AgeQ",
                adjust = c("PA_total_METQ","Sex","Race",

"Education","smoke","alcohol.user","PIR","Hypertension",
                             "DM","HDL_C","LDL_C",
                             "Antihyperlipidemic","Antihyperglycemic",
                             "Antihypertensive","BMIQ","take_drug","CVD"),
                xlsx = "Subgroup01.xlsx")




stratum_model(object= nhs,
```

```
                  y = "OA",
                  x = "CTI",
                  stratum = "Sex",
                  adjust = c("PA_total_METQ","AgeQ","Race",

"Education","smoke","alcohol.user","PIR","Hypertension",
                              "DM","HDL_C","LDL_C",
                              "Antihyperlipidemic","Antihyperglycemic",
                              "Antihypertensive","BMIQ","take_drug","CVD"),
                  xlsx = "Subgroup02.xlsx")



stratum_model(object= nhs,
                  y = "OA",
                  x = "CTI",
                  stratum = "Race",
                  adjust = c("PA_total_METQ","AgeQ","Sex",

"Education","smoke","alcohol.user","PIR","Hypertension",
                              "DM","HDL_C","LDL_C",
                              "Antihyperlipidemic","Antihyperglycemic",
                              "Antihypertensive","BMIQ","take_drug","CVD"),
                  xlsx = "Subgroup03.xlsx")




stratum_model(object= nhs,
                  y = "OA",
                  x = "CTI",
                  stratum = "Education",
                  adjust = c("PA_total_METQ","AgeQ","Sex",
                              "Race","smoke","alcohol.user","PIR","Hypertension",
                              "DM","HDL_C","LDL_C",
                              "Antihyperlipidemic","Antihyperglycemic",
                              "Antihypertensive","BMIQ","take_drug","CVD"),
                  xlsx = "Subgroup04.xlsx")




stratum_model(object= nhs,
                  y = "OA",
```

```
                    x = "CTI",
                    stratum = "smoke",
                    adjust = c("PA_total_METQ","AgeQ","Sex",

"Race","Education","alcohol.user","PIR","Hypertension",
                                   "DM","HDL_C","LDL_C",
                                   "Antihyperlipidemic","Antihyperglycemic",
                                   "Antihypertensive","BMIQ","take_drug","CVD"),
                    xlsx = "Subgroup05.xlsx")


stratum_model(object= nhs,
                    y = "OA",
                    x = "CTI",
                    stratum = "alcohol.user",
                    adjust = c("PA_total_METQ","AgeQ","Sex",
                                   "Race","Education","smoke","PIR","Hypertension",
                                   "DM","HDL_C","LDL_C",
                                   "Antihyperlipidemic","Antihyperglycemic",
                                   "Antihypertensive","BMIQ","take_drug","CVD"),
                    xlsx = "Subgroup06.xlsx")



stratum_model(object= nhs,
                    y = "OA",
                    x = "CTI",
                    stratum = "Hypertension",
                    adjust = c("PA_total_METQ","AgeQ","Sex",
                                   "Race","Education","smoke","PIR","alcohol.user",
                                   "DM","HDL_C","LDL_C",
                                   "Antihyperlipidemic","Antihyperglycemic",
                                   "Antihypertensive","BMIQ","take_drug","CVD"),
                    xlsx = "Subgroup07.xlsx")



stratum_model(object= nhs,
                    y = "OA",
                    x = "CTI",
                    stratum = "DM",
                    adjust = c("PA_total_METQ","AgeQ","Sex",
                                   "Race","Education","smoke","PIR","alcohol.user",
                                   "Hypertension","HDL_C","LDL_C",
```

```
                    "Antihyperlipidemic","Antihyperglycemic",
                    "Antihypertensive","BMIQ","take_drug","CVD"),
          xlsx = "Subgroup08.xlsx")




stratum_model(object= nhs,
          y = "OA",
          x = "CTI",
          stratum = "BMIQ",
          adjust = c("PA_total_METQ","AgeQ","Sex",
                    "Race","Education","smoke","PIR","alcohol.user",
                    "Hypertension","HDL_C","LDL_C",
                    "Antihyperlipidemic","Antihyperglycemic",
                    "Antihypertensive","DM","take_drug","CVD"),
          xlsx = "Subgroup09.xlsx")




stratum_model(object= nhs,
          y = "OA",
          x = "CTI",
          stratum = "PA_total_METQ",
          adjust = c("AgeQ","Sex","Race",

"Education","smoke","alcohol.user","PIR","Hypertension",
                    "DM","HDL_C","LDL_C",
                    "Antihyperlipidemic","Antihyperglycemic",
                    "Antihypertensive","BMIQ","take_drug","CVD"),
          xlsx = "Subgroup10.xlsx")




stratum_model(object= nhs,
          y = "OA",
          x = "CTI",
          stratum = "take_drug",
          adjust = c("AgeQ","Sex","Race",

"Education","smoke","alcohol.user","PIR","Hypertension",
                    "DM","HDL_C","LDL_C",
                    "Antihyperlipidemic","Antihyperglycemic",
```

```
"Antihypertensive","BMIQ","CVD","PA_total_METQ"),
                   xlsx = "Subgroup11.xlsx")




# ROC 曲线
r1 <- svy_roc(design = nhs,score = "CTI",class = "OA",)
svy_roc_plot(r1)


r2 <- svy_roc(design = nhs,score = "TyG",class = "OA",)
svy_roc_plot(r2)


r3 <- svy_roc(design = nhs,score = "CRP",class = "OA",)
svy_roc_plot(r3)

svy_roc_plot(r1,r2,r3,legend.title = "ROC",
                 legend.names = c("CTI(AUC=0.620)","TyG(AUC=0.600)",
                                  "CRP(AUC=0.598)"))
```

```
library(nhanesR)
setwd("C:\\Users\\xiaob\\Desktop\\CTI\\Mort")

d1 <- dex_TyG(years = c(1999:2018))



d2 <- db_demo(years = c(1999:2018),ageyr = "Age",sex = "Sex",eth1 = "Race",
              edu = "Education",
              poverty = "PIR",Year = T) |>
    db_bodyMeasure(years = c(1999:2018),
                   BMI_kg.m2 = "BMI") |>
    db_HemalBiochemistry(years = c(1999:2018),fast_glucose_mg.dl = "FBG",
                         fast_triglyceride_mg.dl = "TG",
                         C_reactive_protein_mg.dl = "CRP",
                         hdl_cholesterol_mg.dl = "HDL_C",
                         ldl_cholesterol_mg.dl = "LDL_C",
                         wtsaf2yr = T,wtsaf4yr = T) |>
    diag_smoke(years = c(1999:2018)) |>
    diag_alcohol.user(years = c(1999:2018)) |>

    diag_Hypertension(years = c(1999:2018)) |>
```

```
        diag_DM(years = c(1999:2018))


d4 <- diag_arthritis(years = c(1999:2018),arthritis = T,arghritis_type = T,
                              osteoarthritis_or_degenerative_arthritis = T)




d0 <- Left_Join(d2,d1)

d0 <- Left_Join(d0,d4)

write.xlsx(x = d0,file = "d0.xlsx")


d0 <- drop_row(d0,is.na(d0$CRP))
d0 <- drop_row(d0,is.na(d0$TyG))
missValue(d0)
d0$CTI <- 0.412 * ifelse(d0$CRP > 0, log(d0$CRP), NA) + d0$TyG


write.xlsx(x = d0,file = "d8.xlsx")



d5 <- read.xlsx("d9.xlsx")


d3<-dex_PhysicalActivity(years = (1999:2018),all.5 = TRUE,
                                  MET=TRUE,total_MET=TRUE) |>
        drug_anti.Hyperlipidemic(years = c(1999:2018),
                                      take_drug = "Antihyperlipidemic") |>
        drug_anti.Diabetic(take_drug = "Antihyperglycemic")|>
        drug_anti.Hypertensive(take_drug = "Antihypertensive")
d3 <- d3 %>%
        mutate(PA_total_METQ = ifelse(PA_total_MET < 600, "<600",
                                           ifelse(PA_total_MET      >=      600      ,
"≥600",NA)))
d3 <- drop_col(d3,"PA_total_MET")


d6 <- drug_search("glucocorticoids",years =c(1999:2018) )
```

```
d6 <- Drug("glucocorticoids",years = c(1999:2018) )



d5 <- Left_Join(d5,d3)

d5 <- Left_Join(d5,d6)



d6 <- d5 %>%
    mutate(nhs_wt = case_when(
        Year >= 1990 & Year <= 2000 ~ wtsaf4yr * 2 / 10,
        Year >= 2001 & Year <= 2002 ~ wtsaf4yr * 2 / 10,
        Year >= 2003 & Year <= 2004 ~ wtsaf2yr * 1 / 10,
        Year >= 2005 & Year <= 2006 ~ wtsaf2yr * 1 / 10,
        Year >= 2007 & Year <= 2008 ~ wtsaf2yr * 1 / 10,
        Year >= 2009 & Year <= 2010 ~ wtsaf2yr * 1 / 10,
        Year >= 2011 & Year <= 2012 ~ wtsaf2yr * 1 / 10,
        Year >= 2013 & Year <= 2014 ~ wtsaf2yr * 1 / 10,
        Year >= 2015 & Year <= 2016 ~ wtsaf2yr * 1 / 10,
        Year >= 2017 & Year <= 2018 ~ wtsaf2yr * 1 / 10))



d8 <- d6[d6$nhs_wt != 0, ]



d9 <- drop_col(d8,"wtsaf2yr","wtsaf4yr","arthritis","arghritis_type","Year",

               "osteoarthritis_or_degenerative_arthritis")
summary(d9)


# Recode(d9$Race)
d9$Race <- Recode(d9$Race,
                  "Non-Hispanic White::",
                  "Non-Hispanic Black::",
                  "Mexican American::",
                  "Other Hispanic::",
                  "Other Race - Including Multi-Racial::Other Race",
                  to.numeric = FALSE)
# Recode(d9$Education)
```

```
d9$Education <- Recode(d9$Education,
                       "College Graduate or above::College or above",
                       "9-11th Grade (Includes 12th grade with no diploma)::Less than high school",
                       "High School Grad/GED or Equivalent::High school or equivalent",
                       "Some College or AA degree::College or above",
                       "Less Than 9th Grade::Less than high school",
                       "NA::",
                       to.numeric = FALSE)

# Recode(d9$DM)
d9$DM <- Recode(d9$DM,
                "no::no",
                "DM::yes",
                "IFG::no",
                "IGT::no",
                "NA::",
                to.numeric = FALSE)
# Recode(d9$Antihyperlipidemic)
d9$Antihyperlipidemic <- Recode(d9$Antihyperlipidemic,
                                "other::no",
                                "no::no",
                                "yes::yes",
                                "NA::",
                                to.numeric = FALSE)
# Recode(d9$Antihyperglycemic)
d9$Antihyperglycemic <- Recode(d9$Antihyperglycemic,
                               "other::no",
                               "no::no",
                               "yes::yes",
                               "NA::",
                               to.numeric = FALSE)
# Recode(d9$Antihypertensive)
d9$Antihypertensive <- Recode(d9$Antihypertensive,
                              "yes::",
                              "no::",
                              "other::no",
                              "NA::",
                              to.numeric = FALSE)
# Recode(d9$take_drug)
d9$take_drug <- Recode(d9$take_drug,
                       "yes::",
                       "no::",
```

```
                                   "other::no",
                                   "NA::",
                                   to.numeric = FALSE)



d2 <- diag_CVD(years = c(1999:2018))
d9 <- Left_Join(d9,d2)




d9 <- d9 %>%
    mutate(BMIQ = ifelse(BMI < 25, "<25",
                         ifelse(BMI >= 25 , "≥25",NA)))   #  处理缺失值
d9 <- d9 %>%
    mutate(AgeQ = ifelse(Age < 65, "<65",
                         ifelse(Age >= 65 , "≥65",NA)))   #  处理缺失值
d9$CTIQ <- cut(
    d9$CTI,
    breaks = quantile(d9$CTI, probs = c(0, 0.25, 0.5, 0.75, 1)),
    labels = c("Q1", "Q2", "Q3", "Q4"),
    include.lowest = TRUE
)

quantile_values1 <- quantile(
    d9$CTI,
    probs = c(0, 0.25, 0.5, 0.75, 1)
)
print(quantile_values1)



d3 <- db_mort(years = c(1999:2018))
Left_Join(d9,d3)
d2 <- Left_Join(d9,d3)

table(d2$eligstat)
table(d2$mortstat,useNA = "i")
d2 <- select_row(d2,d2$eligstat=="Eligible")

unique(d2$mortstat)
unique(d2$ucod_leading)
table(d2$ucod_leading) |> do::decrease()
```

```
head(d)

d2 <- drop_col(d2,"eligstat","diabetes","hyperten","permth_int")

col_rename(d2) <- c("mortstat:status","ucod_leading:leading","permth_exm:time")

table(d2$status)

# Recode(d2$status)
d2$status <- Recode(d2$status,
                    "Assumed deceased::1",
                    "Assumed alive::0",
                    to.numeric = TRUE)
# Recode(d2$leading)
d2$leading <- Recode(d2$leading,
                     "Diseases of heart (I00-I09, I11, I13, I20-I51)::Heart",
                     "Malignant neoplasms (C00-C97)::Cancer",
                     "All other causes (residual)::Other",
                     "Influenza and pneumonia (J09-J18)::Influenza",
                     "Accidents (unintentional injuries) (V01-X59, Y85-Y86)::Accidents",
                     "Chronic lower respiratory diseases (J40-J47)::Lung",
                     "Diabetes mellitus (E10-E14)::Diabetes",
                     "Alzheimer's disease (G30)::Alzheimer",
                     "Cerebrovascular diseases (I60-I69)::Cerebrovascular",
                     "Nephritis, nephrotic syndrome and nephrosis (N00-N07, N17-N19, N25-N27)::Kidney",
                     "NA::",
                     to.numeric = FALSE)


d2 <- add_col(data = d2,colname = "leading",value = "no",
              condition = is.na(d2$leading))



d2 <- subset(d2, OA != 0)

d2$time <- as.numeric(as.character(d2$time))


library(missRanger)
set.seed(123)
data.imp<- missRanger(d2)
```

```
library(survey)
nhs <- svy_design(data.imp)



svy_tableone(design = nhs,cv = c("CTI","Age","PIR", "FBG",
                                  "TG","TyG","HDL_C","LDL_C",
                                  "BMI","CRP"),
             c_meanPMse = T,
             gv = c("Sex","Race","Education","smoke",
                    "alcohol.user","Hypertension",
                    "DM","Antihyperlipidemic","Antihyperglycemic",
                    "Antihypertensive","CVD","take_drug","PA_total_METQ"),
             g_nSQper = T,round = 2,by = "status",xlsx = "tablestatus1.xlsx")

svy_tableone(design = nhs,cv = c("CTI","Age","PIR", "FBG",
                                  "TG","TyG","HDL_C","LDL_C",
                                  "BMI","CRP"),
             c_meanPMse = T,
             gv = c("Sex","Race","Education","smoke",
                    "alcohol.user","Hypertension",
                    "DM","Antihyperlipidemic","Antihyperglycemic",
                    "Antihypertensive","CVD","take_drug","PA_total_METQ"),
             g_nSQper = T,round = 2,xlsx = "tablestatusall1.xlsx")



s1 <- svykm(Surv(time,status) ~CTIQ,design = nhs)
svy_kmplot(s1,risktable = F,ci = F,legend.title = "CTI")
```

```
f0 <- svycoxph(Surv(time,status)~CTI,nhs) |> reg_table()
f1 <- svycoxph(Surv(time,status)~CTI+Age+Sex+Race,nhs) |> reg_table()
f2 <- svycoxph(Surv(time,status)~CTI+Age+Sex+Race+Education+

smoke+alcohol.user+PIR+Hypertension+DM+BMI+HDL_C+LDL_C+
                              CVD+PA_total_METQ+take_drug+Antihyperlipidemic+
                              Antihyperglycemic+Antihypertensive,nhs) |> reg_table()
crude.Model.n(f0,f1,f2,style = 1,xlsx = "CTI.xlsx")



f0 <- svycoxph(Surv(time,status)~CTIQ,nhs) |> reg_table()
f1 <- svycoxph(Surv(time,status)~CTIQ+Age+Sex+Race,nhs) |> reg_table()
f2 <- svycoxph(Surv(time,status)~CTIQ+Age+Sex+Race+Education+

smoke+alcohol.user+PIR+Hypertension+DM+BMI+HDL_C+LDL_C+
                              CVD+PA_total_METQ+take_drug+Antihyperlipidemic+
                              Antihyperglycemic+Antihypertensive,nhs) |> reg_table()
crude.Model.n(f0,f1,f2,style = 1,xlsx = "CTIQ.xlsx")



library(rms)
f8 <- svycoxph(Surv(time,status)~rcs(CTI,4)+Age+Sex+Race+Education+

smoke+alcohol.user+PIR+Hypertension+DM+BMI+HDL_C+LDL_C+
                              CVD+PA_total_METQ+take_drug+Antihyperlipidemic+
                              Antihyperglycemic+Antihypertensive,
                        design = nhs)

optimal_nKnots(f8)
r8 <- RCS(f8)
ggplot(r8)
```

```
getChangepoints(r8)

stratum_model(object= nhs,
              y = "status",
              x = "CTI",
              stratum = "Sex",
              adjust = c("BMIQ","PA_total_METQ","Race",

"Education","smoke","alcohol.user","PIR","Hypertension",
                         "DM","HDL_C","LDL_C",
                         "Antihyperlipidemic","Antihyperglycemic",
                         "Antihypertensive","AgeQ","take_drug","CVD"),
              xlsx = "Subgroup1.xlsx",round = 2)

stratum_model(object= nhs,
              y = "status",
              x = "CTI",
              stratum = "Race",
              adjust = c("BMIQ","PA_total_METQ","Sex",

"Education","smoke","alcohol.user","PIR","Hypertension",
                         "DM","HDL_C","LDL_C",
                         "Antihyperlipidemic","Antihyperglycemic",
                         "Antihypertensive","AgeQ","take_drug","CVD"),
              xlsx = "Subgroup2.xlsx",round = 2)

stratum_model(object= nhs,
              y = "status",
              x = "CTI",
```

```
                    stratum = "Education",
                    adjust = c("BMIQ","PA_total_METQ","Race",
                               "Sex","smoke","alcohol.user","PIR","Hypertension",
                               "DM","HDL_C","LDL_C",
                               "Antihyperlipidemic","Antihyperglycemic",
                               "Antihypertensive","AgeQ","take_drug","CVD"),
                    xlsx = "Subgroup3.xlsx",round = 2)



stratum_model(object= nhs,
                    y = "status",
                    x = "CTI",
                    stratum = "smoke",
                    adjust = c("BMIQ","PA_total_METQ","Race",

"Sex","Education","alcohol.user","PIR","Hypertension",
                               "DM","HDL_C","LDL_C",
                               "Antihyperlipidemic","Antihyperglycemic",
                               "Antihypertensive","AgeQ","take_drug","CVD"),
                    xlsx = "Subgroup4.xlsx",round = 2)



stratum_model(object= nhs,
                    y = "status",
                    x = "CTI",
                    stratum = "alcohol.user",
                    adjust = c("BMIQ","PA_total_METQ","Race",
                               "Sex","Education","smoke","PIR","Hypertension",
                               "DM","HDL_C","LDL_C",
                               "Antihyperlipidemic","Antihyperglycemic",
                               "Antihypertensive","AgeQ","take_drug","CVD"),
                    xlsx = "Subgroup5.xlsx",round = 2)



stratum_model(object= nhs,
                    y = "status",
                    x = "CTI",
                    stratum = "Hypertension",
                    adjust = c("BMIQ","PA_total_METQ","Race",
                               "Sex","Education","smoke","PIR","alcohol.user",
                               "DM","HDL_C","LDL_C",
```

```
                       "Antihyperlipidemic","Antihyperglycemic",
                       "Antihypertensive","AgeQ","take_drug","CVD"),
           xlsx = "Subgroup6.xlsx",round = 2)


stratum_model(object= nhs,
           y = "status",
           x = "CTI",
           stratum = "DM",
           adjust = c("BMIQ","PA_total_METQ","Race",
                       "Sex","Education","smoke","PIR","alcohol.user",
                       "Hypertension","HDL_C","LDL_C",
                       "Antihyperlipidemic","Antihyperglycemic",
                       "Antihypertensive","AgeQ","take_drug","CVD"),
           xlsx = "Subgroup7.xlsx",round = 2)


stratum_model(object= nhs,
           y = "status",
           x = "CTI",
           stratum = "BMIQ",
           adjust = c("DM","PA_total_METQ","Race",
                       "Sex","Education","smoke","PIR","alcohol.user",
                       "Hypertension","HDL_C","LDL_C",
                       "Antihyperlipidemic","Antihyperglycemic",
                       "Antihypertensive","AgeQ","take_drug","CVD"),
           xlsx = "Subgroup8.xlsx",round = 2)


stratum_model(object= nhs,
           y = "status",
           x = "CTI",
           stratum = "AgeQ",
           adjust = c("DM","PA_total_METQ","Race",
                       "Sex","Education","smoke","PIR","alcohol.user",
                       "Hypertension","HDL_C","LDL_C",
                       "Antihyperlipidemic","Antihyperglycemic",
                       "Antihypertensive","BMIQ","take_drug","CVD"),
           xlsx = "Subgroup9.xlsx",round = 2)
```

```
stratum_model(object= nhs,
                     y = "status",
                     x = "CTI",
                     stratum = "PA_total_METQ",
                     adjust = c("BMIQ","Sex","Race",

"Education","smoke","alcohol.user","PIR","Hypertension",
                                     "DM","HDL_C","LDL_C",
                                     "Antihyperlipidemic","Antihyperglycemic",
                                     "Antihypertensive","AgeQ","take_drug","CVD"),
                     xlsx = "Subgroup10.xlsx",round = 2)



stratum_model(object= nhs,
                     y = "status",
                     x = "CTI",
                     stratum = "take_drug",
                     adjust = c("BMIQ","Sex","Race",

"Education","smoke","alcohol.user","PIR","Hypertension",
                                     "DM","HDL_C","LDL_C",
                                     "Antihyperlipidemic","Antihyperglycemic",

"Antihypertensive","AgeQ","PA_total_METQ","CVD"),
                     xlsx = "Subgroup11.xlsx",round = 2)
```
